# Consciousness in humans and non-human animals: recent advances and future directions

**DOI:** 10.3389/fpsyg.2013.00625

**Published:** 2013-10-31

**Authors:** Melanie Boly, Anil K. Seth, Melanie Wilke, Paul Ingmundson, Bernard Baars, Steven Laureys, David B. Edelman, Naotsugu Tsuchiya

**Affiliations:** ^1^Department of Neurology, University of WisconsinMadison, WI, USA; ^2^Department of Psychiatry, Center for Sleep and Consciousness, University of WisconsinMadison, WI, USA; ^3^Coma Science Group, Cyclotron Research Centre and Neurology Department, University of Liege and CHU Sart Tilman HospitalLiege, Belgium; ^4^Sackler Centre for Consciousness Science, University of SussexBrighton, UK; ^5^Department of Informatics, University of SussexBrighton, UK; ^6^Department of Cognitive Neurology, University Medical School GoettingenGoettingen, Germany; ^7^German Primate Center, Leibniz Institute for Primate ResearchGoettingen, Germany; ^8^Mind Science FoundationSan Antonio, TX, USA; ^9^The Neurosciences InstituteLa Jolla, CA, USA; ^10^Science and Mathematics, Bennington CollegeBennington, VT, USA; ^11^Faculty of Medicine, Nursing and Health Sciences, School of Psychology and Psychiatry, Monash UniversityClayton, VIC, Australia; ^12^Decoding and Controlling Brain Information, Japan Science and Technology AgencyChiyoda-ku, Tokyo, Japan

**Keywords:** consciousness, animals, human cognition, theoretical neuroscience, biotechnology, neuroimaging

## Abstract

This joint article reflects the authors' personal views regarding noteworthy advances in the neuroscience of consciousness in the last 10 years, and suggests what we feel may be promising future directions. It is based on a small conference at the Samoset Resort in Rockport, Maine, USA, in July of 2012, organized by the Mind Science Foundation of San Antonio, Texas. Here, we summarize recent advances in our understanding of subjectivity in humans and other animals, including empirical, applied, technical, and conceptual insights. These include the evidence for the importance of fronto-parietal connectivity and of “top-down” processes, both of which enable information to travel across distant cortical areas effectively, as well as numerous dissociations between consciousness and cognitive functions, such as attention, in humans. In addition, we describe the development of mental imagery paradigms, which made it possible to identify covert awareness in non-responsive subjects. Non-human animal consciousness research has also witnessed substantial advances on the specific role of cortical areas and higher order thalamus for consciousness, thanks to important technological enhancements. In addition, much progress has been made in the understanding of non-vertebrate cognition relevant to possible conscious states. Finally, major advances have been made in theories of consciousness, and also in their comparison with the available evidence. Along with reviewing these findings, each author suggests future avenues for research in their field of investigation.

## Introduction

Due to many technical and conceptual advances, the neuroscience of consciousness has witnessed considerable progress over the last decade. During a meeting in summer 2012, we practiced the difficult exercise of synthesizing what we felt were the most outstanding recent advances in the field and what we believed represent promising avenues for further progress. At the conclusion of the meeting, we decided that the outcome of this discussion might be of interest to other researchers in the field, and could potentially provoke stimulating and fruitful dialogue. Recent groundbreaking brain imaging studies have enabled scientists and clinicians to detect the presence of consciousness in some patients who appear to have lost consciousness by existing clinical criteria. Actual loss of consciousness due to various causes is typically accompanied by breakdown of brains' capacity to integrate neuronal activity across distant areas, especially via top-down or reentrant connectivity supported by the integrity of fronto-parietal areas. In normal healthy humans, the contents of consciousness have been examined mainly with psychophysical and neuroimaging techniques, uncovering that consciousness may be neither sufficient nor necessary for high-level cognitive functions, such as attention, cognitive control, and volition, at least in simple form. Non-human animal consciousness research has also witnessed groundbreaking advances in the study of contents of consciousness by employing perceptual rivalry paradigms and elucidating the effect of reversible thalamic and cortical inactivations. We also review recent progress in our knowledge of phylogenetic origins of consciousness through comparative studies of non-vertebrate cognition. Finally, recent years have witnessed major theoretical advances, and signs of convergence between theories and evidence. Given the progress seen in the last decade, the coming years will, no doubt, continue to be exciting times for investigators seeking to deepen our understanding of the neuroscientific basis of conscious experience.

## Advances in consciousness science in humans and animals: a 10-year retrospective

In this section, we will describe what we feel are important advances in consciousness science in the last 10 years. We will first provide a brief overview of advances made in the study of altered levels of consciousness (i.e., those due to brain damage, anesthesia, seizures, or during sleep). We will then discuss some major conceptual and practical advances in the study of neural correlates of conscious contents in healthy awake volunteers (HAVs). Reflecting these two questions, it is critical at the outset to distinguish between the search for neural correlates or underpinnings of conscious *level* and of conscious *content*. This distinction between will recur throughout our discussion. We will also review critical issues and advances in the study of contents of consciousness in non-human animals. Finally, we will discuss several neuroscientific theories that have become increasingly influential over the last decade, focusing on how each individually addresses the problem of consciousness. Due to space limitations, several important topics will not be covered here: (i) the neuroscience of conscious selfhood, including “out-of-body” experiences, and other manipulations of the experience of “body ownership,” (ii) related notions of emotions, intention, volition, agency, and the suggested role of consciousness in social cognition (Frith and Frith, 2007), (iii) disorders of conscious content (such as hallucinations, delusions, and neuropsychiatric conditions generally), (iv) the effects of psychotropic substances on consciousness and (v) changes in conscious contents in meditative, hypnotic, and similar states.

### Altered levels of consciousness in humans

**Figure 1:**
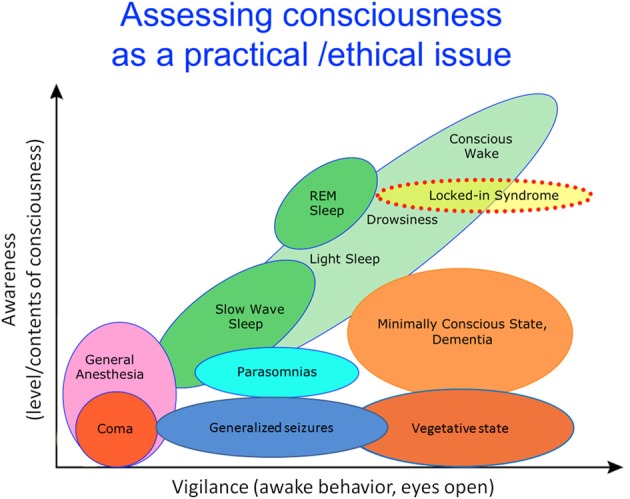
**Level and contents of consciousness.** The level of consciousness can be dissociated from behaviors that are traditionally regarded as a signs of vigilance or arousal (such as opening of eyes, command following etc.). Typically, high conscious levels are associated with an increased range of conscious contents. Whether or not high level of consciousness without any conscious contents is possible remains unclear. Adapted from Laureys (2005), courtesy of Giulio Tononi.

The study of altered levels of consciousness such as coma or anesthesia has provided clinical, ethical and even legal motivations for the neuroscientific study of consciousness (Laureys and Schiff, 2012) (Figure 1). A better understanding of the links between consciousness and the brain is indeed required to better characterize neural markers of consciousness in these states (Boly et al., 2009). An improved understanding could also facilitate new treatments for severely brain-damaged patients (Schiff, 2010). Finally, the issue of awareness under anesthesia merits greater scrutiny, particularly given the evidence that such episodes may commonly be missed by applying the various techniques currently used to monitor brain function under sedation (Avidan et al., 2011).

Recent advances in functional imaging and electrophysiological techniques (encompassing functional MRI, EEG, and intracranial recordings) applied in these contexts have significantly expanded our knowledge of neural correlates of conscious level in humans. Accordingly, some outstanding advances in this field are summarized below.

#### Severe brain damaged patients: coma, vegetative state (VS), and minimally conscious states (MCS)

**Figure 2:**
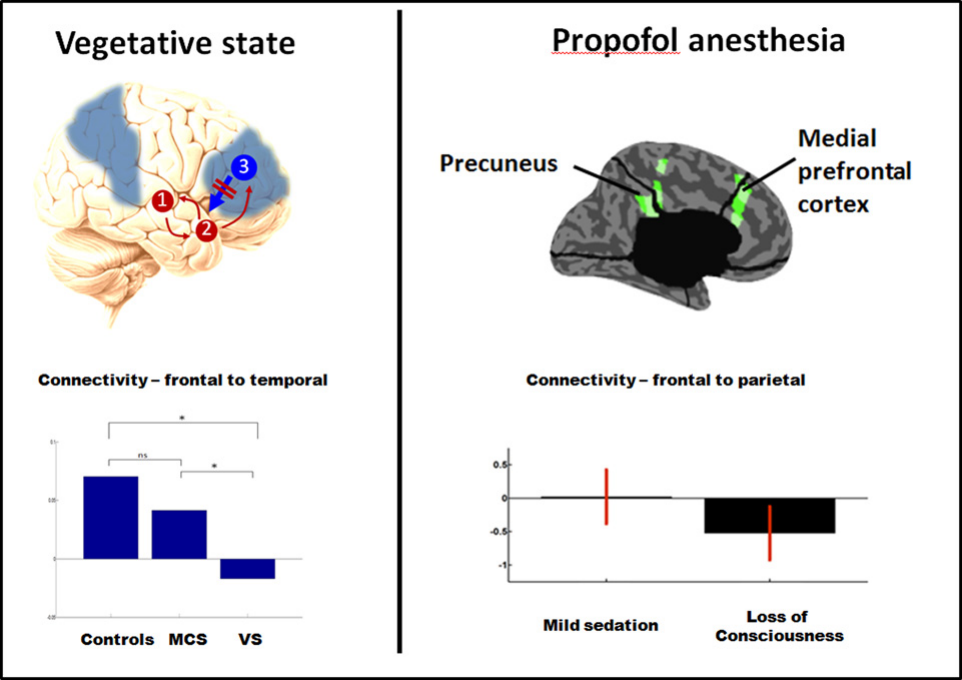
**PET studies reveal hypometabolism in similar fronto-parietal areas in vegetative and anesthesia (areas in blue, left panel).** Recent dynamic causal modeling studies also suggest a loss of top-down (reentrant) connectivity in fronto-parietal cortices in both vegetative state (left panel, assessed for response to auditory stimuli) and propofol anesthesia (right panel, assessed from spontaneous EEG). Adapted from Boly et al. (2011, 2012a). ^*^Significant difference between conditions (corrected *p* < 0.05).

Research on altered states of consciousness due to brain damage has greatly benefited from the definition of the minimally conscious state (MCS), provided by Giacino et al. (2002). Since the introduction of this definition, a number of studies have shown significant differences in brain function between vegetative and minimally conscious state patient populations (Boly, 2011). Brain function in patients in the vegetative state (VS) is very similar to that during sleep and anesthesia, and is characterized by an impaired function of thalamus and fronto-parietal cortical areas (Laureys et al., 2000a,b, 2002; Laureys, 2005). In contrast, brain function in MCS is much closer to that observed in HAVs, with the preservation of functional connectivity (temporal correlation patterns between cortical areas) and activation in frontal and parietal cortical areas (Boly et al., 2004, 2008a; Vanhaudenhuyse et al., 2010; Fernandez-Espejo et al., 2012). What makes conscious patients in MCS appear unconscious like VS patients is the lack of motor functions, including speech, which renders patients non-communicative. In addition to differences in brain activation and functional connectivity, recent electroencephalography (EEG) studies have revealed that MCS patients, in contrast to VS patients, show preserved “top-down” or recurrent processing in higher-order cortical areas (Boly et al., 2011) (Figure 2). Furthermore, the brains of the MCS patients and normal healthy people react to a single pulse of transcranial magnetic stimulation (TMS) in a similar way (Rosanova et al., 2012); TMS-evoked EEG response is variable across trials, spreads across widely distributed cortical areas and reveals much more complicated and sustained dynamics than those evoked in the VS patients' brains (also see Casali et al. (2013) the following section on anesthesia (Ferrarelli et al., 2010) and sleep (Massimini et al., 2005) on this TMS-EEG perturbational approach).

The application of new technologies for the assessment of brain function in patients following severe brain injury has led to increasing refinement and differentiation of the taxonomy of impairments of consciousness (Figure 1). New refinements of clinical definitions are ongoing, with the current classification of patients in MCS as “MCS−” and “MCS+,” depending on the presence or absence of response to commands during clinical examination (Laureys and Schiff, 2012). A recent PET study showed that “MCS+” patients display preserved left-sided fronto-parietal activity as compared to the “MCS-” population (Bruno et al., 2012).

**Figure 3:**
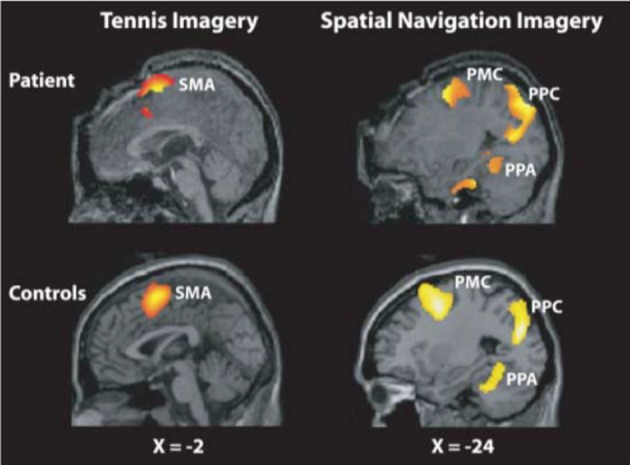
**Mental imagery paradigm used to detect covert awareness in non-communicating patients.** An activation of motor cortex in response to a motor imagery task instruction (“imagine playing tennis,” on the left) or an activation of parahippocampal gyrus in response to the instruction to perform a spatial navigation mental imagery task (“imagine visiting your home,” right) can be considered as evidence of response to command, and thus of the presence of awareness, in patients unresponsive at the bedside. Reproduced with permission from (Owen et al., 2006).

Another revolutionary advance in the field of neuroimaging of non-communicative brain damaged patients has been the design of active paradigms, in which responses to commands are probed while bypassing motor outputs [(Boly et al., 2007b); see Boly and Seth (2012) for a review]. The first paradigms employed in this context probed motor or spatial navigation imagery using functional magnetic resonance imaging (fMRI) (Owen et al., 2006; Monti et al., 2010) (Figure 3). Some EEG equivalents for these paradigms have also been designed to enable communication with patients outside of the MRI-scanner, even at their home (Bekinschtein et al., 2009; Cruse et al., 2011; Scott et al., 2011). Active paradigms typically reveal that about 20% of VS patients can respond to commands in an intentional manner (Monti et al., 2010; Cruse et al., 2011). While those responding patients are likely to be conscious, non-responsive patients in these paradigms might also be conscious, but not recognized as such due to the insensitivity of these methods. Active paradigms (asking the patients to perform a task, even without motor output) indeed heavily rely on the collaboration of the patients, as well as on intact language capabilities. In fact, it has been shown that some MCS or locked-in syndrome patients, who are conscious, can respond to commands at the bedside, however, they occasionally do not show task-relevant brain activation in response to the instruction to perform the task (Bardin et al., 2011). This finding reinforces the need for more specific identification potentially *sufficient* neural correlates of conscious level in order to detect awareness in patients who do not respond to these tasks. Such research would also inform psychophysiological measures of awareness that do not rely on preserved language comprehension and volition in patients (Scott et al., 2011). As we will see later, the criterion of “sufficiency” regarding neural correlates of consciousness is where recent advances in theory are extremely pertinent.

Finally, the last few years have witnessed the emergence of new treatments for patients with disorders of consciousness. For example, medial thalamic stimulation has proven beneficial for the recovery of responsiveness in some MCS patients (Schiff et al., 2007), suggesting an important role for the thalamus in consciousness. Amantadine, a dopaminergic drug (Giacino et al., 2012), and surprisingly zolpidem, a GABAergic agonist (Whyte and Myers, 2009), have also proven beneficial in some cases of VS or MCS. While the foregoing progress is indeed encouraging, considerable research is still needed in this field.

#### Anesthesia

Similar to what is observed in the VS, brain correlates of general anesthesia commonly involve a functional impairment of thalamus and fronto-parietal areas (Alkire and Miller, 2005). While general anesthesia-induced loss of consciousness (LOC) disrupts functional connectivity within fronto-parietal areas and between fronto-parietal areas and the thalamus, it can leave thalamo-cortical connectivity in low-level sensory cortical areas unchanged (Boveroux et al., 2010). The development of clinically applicable electrophysiological markers of consciousness during general anesthesia therefore remains an important goal (Avidan et al., 2011). Recent studies, using respectively, high density EEG (Murphy et al., 2011; Purdon et al., 2013) and intracranial recordings (Lewis et al., 2012), suggest that increased slow wave activity might be one of the most reliable correlates of LOC under general anesthesia, and subsequent recovery of consciousness (Purdon et al., 2013). Nevertheless, further clinical validation of this marker is needed. Recent EEG studies also provide some evidence that, again similar to what is found in the VS, general anesthesia might be characterized by stereotypical responses to transcranial magnetic stimulation (TMS) (Ferrarelli et al., 2010; Casali et al., 2013) and disruption of “top-down” functional connectivity from frontal to sensory areas (Imas et al., 2005; Lee et al., 2009; Boly et al., 2012a). Interestingly, general anesthesia-induced LOC might also be associated with decreased inhibition in response to external stimulation (Haider et al., 2013), and may also be related to increased synchrony and Granger causality (the latter measures temporal causality between time series) in beta (15–25 Hz) and gamma (25–40 Hz) ranges between fronto-parietal midline areas (Murphy et al., 2011; Barrett et al., 2012) (but also see Figure 2B). Note that while increased gamma synchrony may not be key for sustaining the level of consciousness, as mentioned here, may also play a role in encoding some aspects of contents of consciousness, as reviewed later.

#### Sleep

Neural correlates of reduced consciousness during non-rapid eye movement (NREM) sleep obtained using intracranial recordings and fMRI also indicate decreased activity and functional connectivity in fronto-parietal areas (Horovitz et al., 2009; Samann et al., 2011; Boly et al., 2012b; Nobili et al., 2012). Recent intracranial studies in humans further suggest that NREM sleep occurs primarily at a local level, that is, during this state, the majority of correlated neural activity takes place in isolated modules (Nir et al., 2011). Similarly, TMS-EEG studies have shown decreased effective connectivity (causal interactions in response to perturbation, for example, by a single TMS pulse) and stereotypical patterns of neural responses during NREM sleep (Massimini et al., 2005, 2007; Casali et al., 2013). Recently, however, fMRI studies have confirmed that NREM sleep is a dynamic state. Indeed, both sleep slow waves and spindles induce activation of many thalamo-cortical areas (Schabus et al., 2007; Dang-Vu et al., 2008), but decrease the processing of external stimuli (Dang-Vu et al., 2011; Schabus et al., 2012).

Relatively few studies have investigated brain function during rapid eye movement (REM) sleep. In contrast to NREM sleep, REM sleep is associated with a high percentage of dream reports at awakening (Nir and Tononi, 2010) reflecting conscious states. Nonetheless, brains in the REM sleep compared to those in awake state respond less to external stimuli (Wehrle et al., 2007). Nonetheless, it remains unclear why perception of environmental stimuli is lost during REM sleep. A recent study involving lucid dreamers (who can master preserved control over the contents of their dreams) investigated potential neural correlates of conscious content during REM sleep (Dresler et al., 2011), finding as during normal wakefulness, a correlation between motor imagery dream contents and fMRI activation in the motor cortex. Interestingly, normal REM sleep is associated with reduced gamma activity (Voss et al., 2009) and reduced fronto-parietal metabolism (Dresler et al., 2012) as compared to lucid dreaming. At the sleep onset, a recent fMRI study succeeded to decode more refined conscious contents of visual imagery (Horikawa et al., 2013). Future studies are warranted to better understand the neural correlates underlying changes in conscious level among NREM sleep, REM sleep, and normal waking consciousness.

#### Epilepsy

Neural correlates of epilepsy-induced LOC are preferentially located in the thalamus and fronto-parietal areas (Blumenfeld, 2005; Laureys, 2005); but, the various seizure types differ in how these areas are implicated. While absence seizures (with brief suspension of conscious content, but preserved tonus) and other spike-wave seizures, when associated to LOC, provoke a decrease in fronto-parietal activity (Blumenfeld et al., 2004; Englot et al., 2009), the tonic phase of generalized tonic-clonic seizures induces a widespread increase in fronto-parietal metabolism as compared to baseline (Blumenfeld et al., 2003). Several intracranial recordings in humans have also shown that temporal and parietal lobe seizure-induced LOC is associated with a widespread increase in neural synchrony (Arthuis et al., 2009; Lambert et al., 2012). These results suggest that LOC in epilepsy could be induced due to neural hypersynchrony, impairing the differentiation of neuronal activity within and between cortical areas. To date, data concerning the presence of stereotypical activity in response to perturbation (which could provide direct evidence for reduced effective connectivity) during seizure-induced LOC are missing.

#### The search for a common principle among different unconscious states

Is there a common principle underlying loss of consciousness (LOC) induced by variable causes, due to severe brain injury, general anesthesia, sleep, and seizures (Figure 1)? In recent years, efforts have been made to identify potential common neuronal mechanisms for LOC.

***Anatomical connections and minimal activation in the fronto-parietal areas***. As already mentioned, changes in the activation in fronto-parietal areas are commonly observed in states associated with LOC (Baars et al., 2003; Tononi and Koch, 2008; Bor and Seth, 2012) even if the nature of alteration may vary for each cause. Anatomical connections of the fronto-parietal areas (particularly the precuneus) also suggest their critical role in sustaining the level of consciousness. As central hubs of human brain anatomical connectivity (Hagmann et al., 2008; Buckner et al., 2009), these areas have extensive anatomical and functional relationships, supporting many high-level cognitive functions, such as attention and executive functions. Based on these observations, some authors have argued that the integrity of the fronto-parietal networks may be necessary for consciousness to occur (Demertzi et al., 2013).

However, increasing evidence in HAVs (see below) suggests that in humans, many, if not all, cognitive functions that involve fronto-parietal areas can also operate in the absence of reportable conscious perception of the relevant stimuli, even if performance is usually much diminished (Boly and Seth, 2012; van Gaal and Lamme, 2012). Furthermore, the highest level of metabolic activity in awake human subjects was located in the primary visual cortex, not in the fronto-parietal cortical areas (Vaishnavi et al., 2010). This is counter to what might be expected if fronto-parietal areas are assumed to be continuously active for consciousness to occur. It is also possible, however, that intact anatomical connections along with a minimal level of activity in fronto-parietal areas may be sufficient to support consciousness. Further studies are necessary to establish whether or not anatomical integrity and/or functional activation of fronto-parietal areas are necessary or sufficient for consciousness (Boly et al., 2011). An important role for fronto-parietal networks in consciousness is, however, consistent with evidence describing a range of metabolic activity patterns during different tasks (and rest) since what may be important is intact functional connectivity, not metabolic demand *per se*.

***Slow wave activity***. Electrophysiological recordings during both general anesthesia and sleep suggest that slow wave activity (SWA) may be a common mechanism related to LOC (Boly et al., 2009; Murphy et al., 2011; Lewis et al., 2012). Although patterns of electrophysiological activity in VS patients are more heterogeneous, a recent study (Schnakers et al., 2008) using the “bispectral index” (BIS, which, in low values, is highly weighted by the presence of delta activity in the EEG) suggests that EEG slow frequency activity is higher in unresponsive patients in a vegetative state (VS) than in minimally conscious state patients (MCS), presenting non-reflexive behaviors (Figure 1) on average. To our knowledge, the relationship between SWA and LOC during epileptic seizures has not yet been investigated. At any rate, a mere criterion of presence or absence of slow wave activity cannot be sufficient to establish LOC in every case; indeed, during the tonic phase of generalized tonico-clonic seizures, LOC is associated with a high-frequency, low amplitude EEG (Blumenfeld et al., 2003; Blumenfeld, 2005).

***Effective connectivity and long-distance top-down connectivity***. In instances of general anesthesia, sleep, and vegetative state, TMS-EEG studies (Massimini et al., 2005; Rosanova et al., 2012; Casali et al., 2013) suggest that a common mechanism could also be the loss of effective connectivity, possibly induced by the presence of all-or-none bi-stable neuronal firing dynamics (Tononi and Massimini, 2008). During seizures, hypersynchronous activity might also impair the functional differentiation of specialized cortical areas. Finally, some impairment of functional integrity of top-down (from the anterior to the posterior part of the brain) connections has been suggested during general anesthesia and VS (e.g., Boly et al., 2011, 2012a). Changes in top-down connectivity during seizures and sleep still remain to be tested. We review the relevant evidence in more details in the following sections.

### Contents of consciousness in humans

#### Definitions and measures for contents of consciousness

Studies of neural correlates of consciousness in HAVs often target neural correlates of conscious contents, while the level of consciousness is assumed to be constant. Classically, measures of conscious perception in awake humans distinguish objective performance, such as the ability to discriminate the presence, absence, or identity of distinct stimuli, from subjective reports, such as subjective ratings of the visibility of stimuli or confidence ratings of the accuracy of perceptual decisions, which are associated with conscious perception. Studies taking this approach have benefited from the application of “signal detection theory” (SDT), which provides robust methods for distinguishing objective performance from subjective performance (Maniscalco and Lau, 2012; Barrett et al., 2013), independently of response biases. While subjective ratings are typically obtained by asking subjects to rate their confidence in their responses, several alternatives exist [e.g., post-decision wagering, etc. see Seth et al. (2008) for a review]. Currently there is no consensus about a single best approach, and different measures show empirical as well as theoretical divergence (Overgaard and Sandberg, 2012).

Importantly, a range of studies have outlined dissociations between objective performance and subjective reports, for example in so-called “blindsight” patients [who after lesions of primary visual cortex, lose consciously reportable vision (Persaud et al., 2011)] and in normal subjects (Lau and Passingham, 2006; Sandberg et al., 2011). As such, a prominent experimental strategy when analyzing NCCs of conscious contents is to dissociate objective from subjective responses while measuring neural signals. A selection of the most relevant studies taking this approach is summarized below. Future studies extending this approach as well to patients face a number of important challenges. These include distinguishing between the neuronal correlates of conscious contents *per se* and the neural prerequisites for, and consequences of, conscious states (Aru et al., 2012; de Graaf et al., 2012), as well as (to the extent that they differ) the neural underpinnings of conscious access from those underlying phenomenal consciousness (Block, 2005; Lamme, 2010; Dehaene and Changeux, 2011)—though we acknowledge that the latter distinction remains controversial and much debated (Kouider et al., 2012).

#### Neuroimaging studies of the neural correlates of consciousness of conscious contents

Over the last decades, many studies have investigated NCCs of conscious contents in HAVs. A common paradigm is to employ functional neuroimaging experiments contrasting brain responses to intensity-matched perceived and unperceived stimuli.

**Figure 4:**
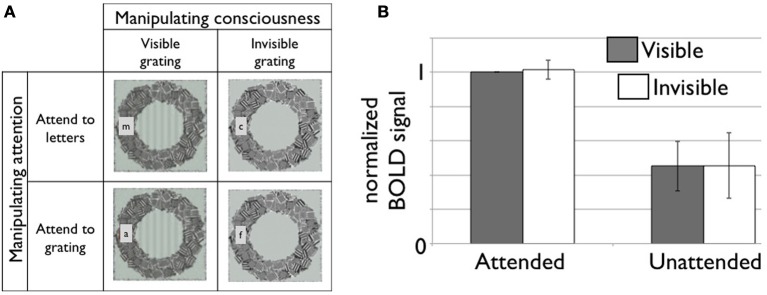
**(A)** A 2-by-2 factorial design for independent manipulation of top-down attention and conscious visibility of the stimulus (Watanabe et al., 2011). Subjects are asked to carry out one of two attention tasks while viewing either a visible or invisible target stimulus. At the same time, dependent variables, such as hemodynamic responses in the brain, are measured. Here, we illustrate roughly what participants perceived in each condition (not the physical stimulus) in the study by Watanabe and colleagues. Subjects either had to report the presence of a target letter when it appeared or whether they could see the target grating or not. **(B)** fMRI responses in V1 is strongly modulated by top-down attention but not by conscious visibility of the grating. Modified based on Figures 2, S2 (*n* = 7 in total) in (Watanabe et al., 2011). The data was provided by the original author. The area under the curve (7–18 s from the block onset) is normalized to the attended and visible condition. The error bar represents 95% confidence interval.

Functional MRI studies typically reveal poor correlation between conscious contents and activity in primary sensory cortical areas (for review see Dehaene and Changeux, 2011). However, the evidence remains mixed. For example, in a bistable perception paradigm (where for the same stimulus, perception alternates between two conscious scenes), activity in primary visual cortex (V1) and lateral geniculate nuclei (LGN) was found to correlate well with conscious perception (Tong, 2003; Haynes and Rees, 2005; Wunderlich et al., 2005). Ongoing discussions concern whether these effects are partially or entirely (Watanabe et al., 2011) confounded by the effects of attention (Boynton, 2011; Watanabe et al., 2011) (Figure 4). It is also possible that BOLD signals measured with fMRI may not reflect the underlying spiking activities in the measured area (Maier et al., 2008) (see ***NCC studies in non-human primates***).

More generally, consciousness-related activations are frequently found in higher-order sensory areas (e.g., Tong et al., 1998; Grill-Spector et al., 2000) and in bilateral parietal and prefrontal cortical areas (Dehaene et al., 2001; Lau and Passingham, 2006; Boly et al., 2007a; Farrer et al., 2008; Desmurget and Sirigu, 2009; Sadaghiani et al., 2009; Bor and Seth, 2012; but also see Tse et al., 2005; Tallon-Baudry, 2011).

Event-related potentials (ERPs) and magneto-encephalography (MEG) studies point to long-latency ERP components, possibly involving fronto-parietal areas, as a reliable neuronal marker for conscious perception in healthy controls (Del Cul et al., 2007; Dehaene and Changeux, 2011). Indeed, several studies showed preserved early latency scalp-ERP activity during processing of non-conscious sensory stimuli (Vogel et al., 1998; Sergent et al., 2005). Perhaps the most consistent findings from EEG/MEG studies of the content-related NCCs consist in late (300–500 ms) activity, often represented by a positive component called P3b (van Aalderen-Smeets et al., 2006; Del Cul et al., 2007; Koivisto and Revonsuo, 2008; Bekinschtein et al., 2009; Koivisto et al., 2009). The generators of the P3b ERP have been suggested to involve temporal, parietal, and frontal association areas (Bekinschtein et al., 2009; Mantini et al., 2009).

Conscious perception is also accompanied by increases in power and synchrony in the gamma band (>30 Hz) (Schurger et al., 2006; Melloni et al., 2007; Doesburg et al., 2009; Wyart and Tallon-Baudry, 2009). A small number of studies have, however, also found fronto-parietal activation and gamma activity during unconscious processing of sensory stimuli (Diaz and McCarthy, 2007; Luo et al., 2009). In the alpha and low beta bands (10–20 Hz), long-distance phase synchrony shows consistent changes for consciousness-related activity (Gross et al., 2004; Gaillard et al., 2009; Hipp et al., 2011).

#### Dissociation of conscious perception and high-level cognitive processing

Another highlight of consciousness research during the last 10 years has been the accumulation of evidence for a dissociation between consciousness and many high-level cognitive functions (reviewed in Boly and Seth, 2012; van Gaal and Lamme, 2012). In particular, the development of the continuous flash suppression paradigm (Tsuchiya and Koch, 2005; Tsuchiya et al., 2006), together with traditional backward and meta-contrast masking (Kouider and Dehaene, 2007), has generated considerable new evidence extending the limits of non-conscious processing in the healthy awake human brain. Presently, a number of studies suggest that conscious perception may not be necessary for the operation of various complex cognitive processes, such as attention (Koch and Tsuchiya, 2007), cognitive control (van Gaal et al., 2011), conflict monitoring (van Gaal et al., 2010), volition (Soon et al., 2008), arithmetic (Sklar et al., 2012), or feature binding (Mudrik et al., 2011), and semantic analysis (Kouider and Dehaene, 2007; but also see Kang et al., 2011).

As alluded in the last part of section Altered Levels of Consciousness in Humans, these findings also have relevance for probing the level of consciousness in neurological patients, in a sense that probing one particular cognitive function (e.g., attention or volitional responses) in a patient may not be sufficient to assure that the patient has a high level of consciousness (Boly and Seth, 2012). It is important to remember, however, that the effect size of these complex cognitive processes in the absence of consciousness is typically much smaller, compared to that obtained in the presence of consciousness (van Gaal and Lamme, 2012).

#### Partial awareness

Another important concept that has attracted attention in recent years is that of “partial awareness” (Kouider and Dupoux, 2004; Kouider et al., 2010). The gradual vs. “all-or-none” nature of consciousness has remained controversial (Sergent and Dehaene, 2004; Grill-Spector and Kanwisher, 2005). But, recent refinements in the subjective assessments of conscious perception have led to the recognition of a “gray zone,” in which subjects report that they perceived a stimulus, without always being completely aware of its details, in the sense of being able to provide accurate reports regarding these details (see e.g., Overgaard et al., 2006). Studies using visual crowding have also indicated different levels of subjective visibility of a detectable object (Kouider and Dupoux, 2004; Grill-Spector and Kanwisher, 2005; Pelli and Tillman, 2008; Kouider et al., 2010). Interestingly, crowding phenomena have been well-approximated and to some extent explained by computer vision algorithms that incorporate “statistical perception” in peripheral vision (Freeman and Simoncelli, 2011; Rosenholtz et al., 2012). Partial awareness is associated with intermediate performance levels as compared to no perception or full perception scores (Sandberg et al., 2011). Furthermore, while studies in HAVs typically emphasize fronto-parietal activity for full conscious access to stimuli (Dehaene and Changeux, 2011), two recent studies using continuous flash suppression have found that brain activation related to partial awareness was more evident in associative visual cortical areas (Hesselmann and Malach, 2011; Hesselmann et al., 2011).

#### Spontaneous brain activity and conscious perception

An emerging topic in consciousness research is the influence of spontaneous brain activity fluctuations on the perception of external stimuli (Boly et al., 2008b; Busch et al., 2009; Mathewson et al., 2009). For example, the activity in the lateral fronto-parietal areas and default-mode network seems to have facilitating and suppressive effects, respectively, on conscious perception of somatosensory stimuli (Boly et al., 2007a). For the auditory modality, however, high activity in the lateral fronto-parietal cortical areas seems to have a suppressive effect, while high activity in the default-mode network seems facilitating (Sadaghiani et al., 2009). In a separate line of research, fMRI studies of human subjects under the influence of psylocibin (a psychedelic compound) showed reduced functional connectivity between prefrontal and cingulate cortical areas in task-free conditions (Carhart-Harris et al., 2012). While research on the link between spontaneous brain activity fluctuations and conscious perception is ongoing (Sadaghiani et al., 2010), multiple studies have now demonstrated that amplitude and phase of ongoing neural oscillations at which a visual stimulus is presented, influences evoked cortical activation patterns and perceptual performance (Mathewson et al., 2009; Vanrullen et al., 2011; Chakravarthi and Vanrullen, 2012). These and other studies stress the dynamic character of the brain's responsiveness to sensory stimulation.

### Animal studies

#### Animal models of consciousness

Consciousness research using non-human animals can be divided into two categories. The first category assumes the presence of consciousness in a particular animal species, such as non-human primates, and uses them as a model for human consciousness. The second category attempts to establish whether a particular animal species has conscious experiences or not, in order to understand the phylogenetic origins and comparative physiological basis of consciousness (Edelman and Seth, 2009; Feinberg and Mallatt, 2013; Mashour and Alkire, 2013).

#### NCC studies in non-human primates

Old world monkeys such as rhesus macaques have many similarities in psychophysical performance with humans (Smith et al., 2009; Lynn and Curran, 2010). At the same time, anatomical and functional organization of their brains share many similarities with that of human brains (Orban et al., 2004; Vincent et al., 2007). Therefore, they are frequently used as models for consciousness in humans. Some animal studies of NCCs of altered conscious level (e.g., during general anesthesia or sleep) have already been briefly discussed above. Thus, this section will focus on animal studies of the neural correlates of conscious contents.

Since vision is a dominant sensory modality for both humans and non-human primates, many studies have focused on elucidating the neural substrates of normal and impaired conscious visual perception. Specifically, monkeys have been used to investigate the NCCs of conscious content on the level of single neurons and their populations, since the requisite intracranial microelectrode recordings can only be performed in humans under exceptional circumstances, for example to localize seizure foci in epileptic patients (Kreiman et al., 2002; Mukamel and Fried, 2012). In addition, recent years have seen an increase of studies that combine local, reversible lesions in monkeys with neuroimaging methods in order to further our understanding of the *causal* contribution of certain brain structures to consciousness, as well as study network impairments underlying visual awareness disorders such as blindsight (Yoshida et al., 2008; Schmid et al., 2010; Leopold, 2012), spatial neglect (Wardak et al., 2002a; Gerits et al., 2012; Wilke et al., 2012), and even confidence in visual perception (Komura et al., 2013).

**Figure 5:**
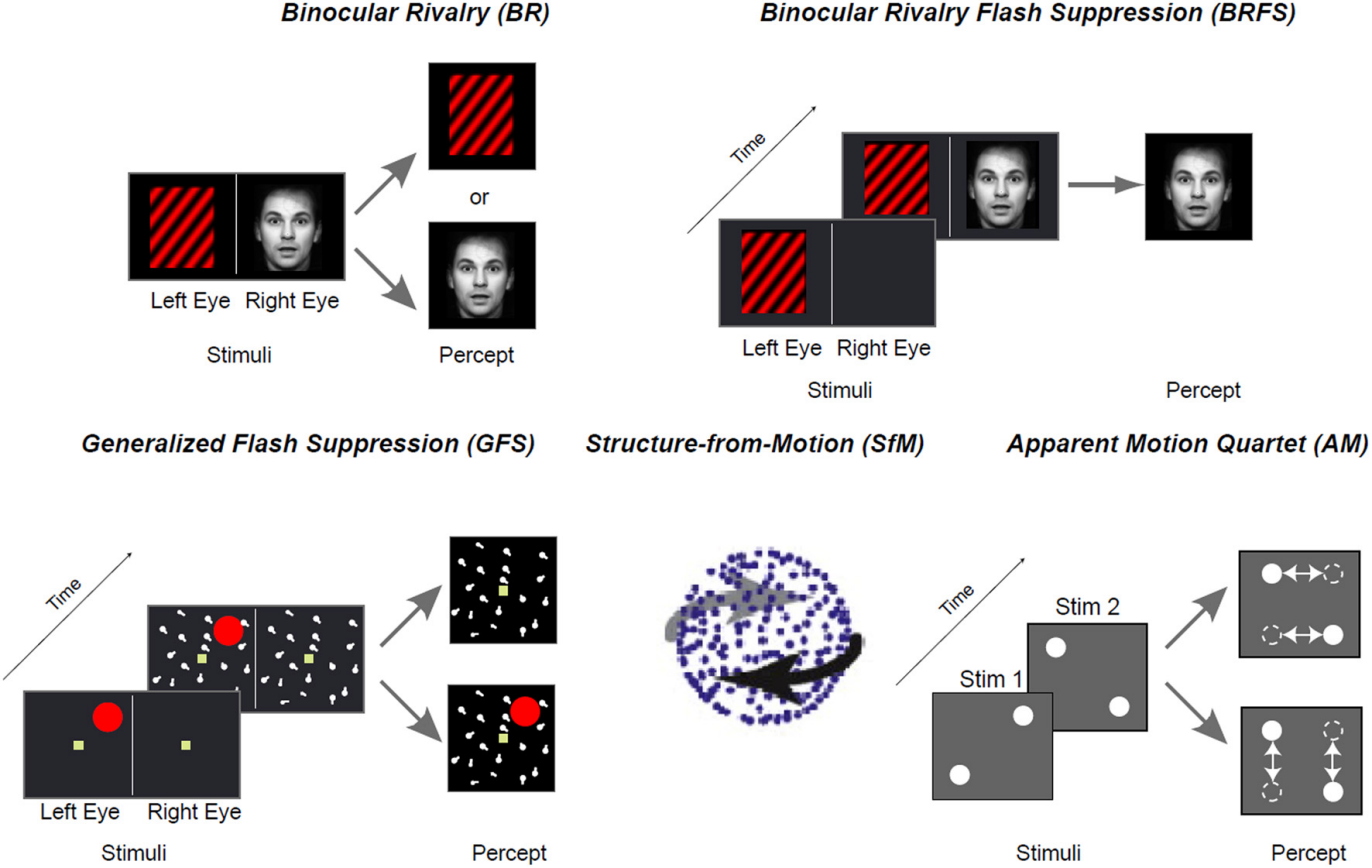
**Examples for bistable stimuli used to investigate the neurophysiological correlates of conscious perception in monkeys**.

**Figure 6:**
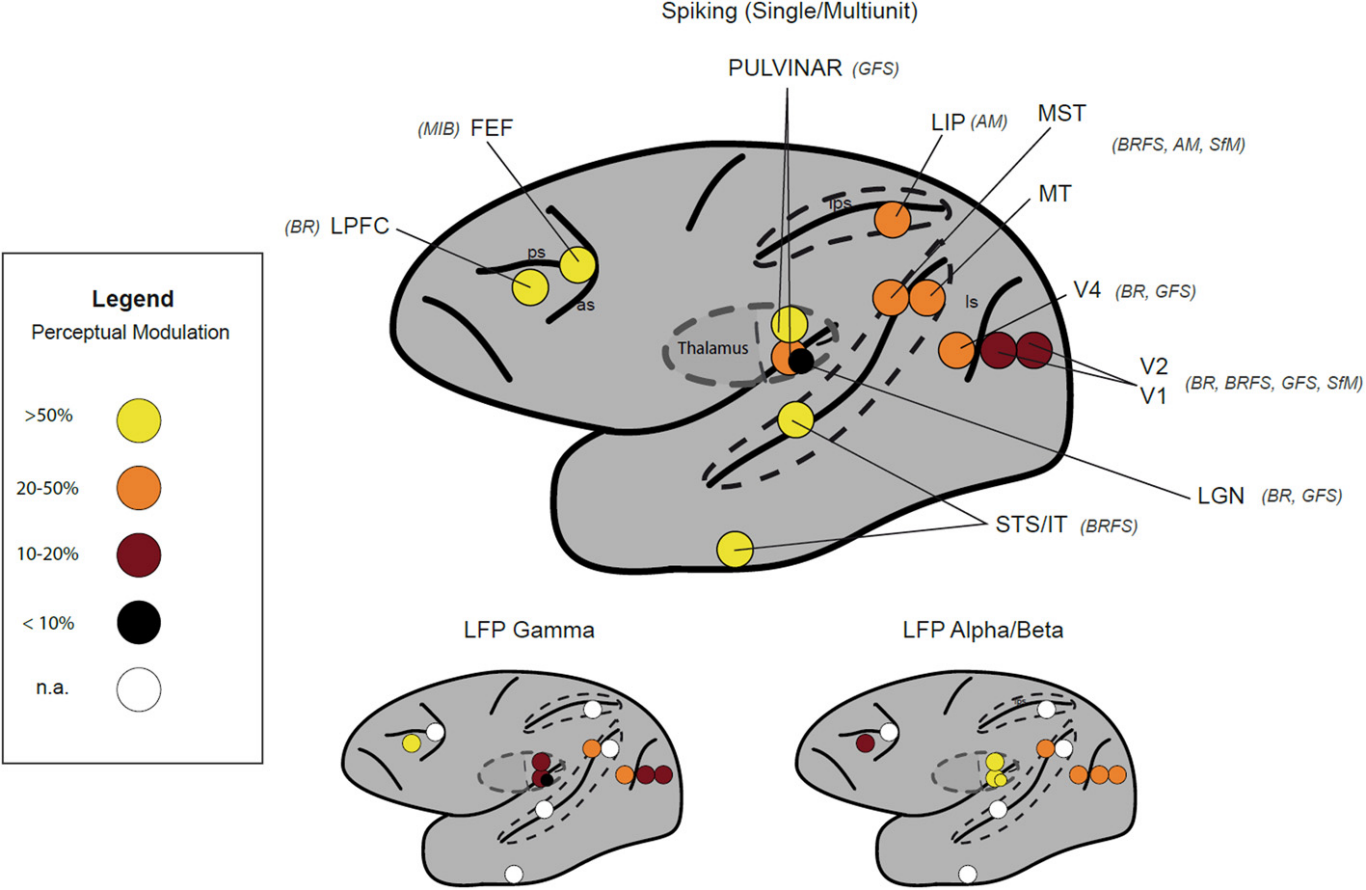
**Neurophysiological correlates of visual awareness measured in different studies by intracranial recordings in monkeys and humans.** Color code depicts the reported percentage of sites modulated for a given signal: Single Unit/Multiunit (upper row), Local Field Potentials (LFP) (lower row) Gamma band (40–100 Hz), Alpha/Beta (8–30 Hz). Stimulus paradigms used in the depicted studies are in brackets next to the area label (see Figure 5). Unavailable information for a given area is given as white circle. Data were derived from following publications: V1-V4 (Leopold and Logothetis, 1996; Gail et al., 2004; Wilke et al., 2006; Maier et al., 2008; Keliris et al., 2010), LGN (Lehky and Maunsell, 1996; Wilke et al., 2009), STS/IT (Sheinberg and Logothetis, 1997; Kreiman et al., 2002), MT/MST (Logothetis and Schall, 1989; Williams et al., 2003; Maier et al., 2007; Wang et al., 2009), LIP (Williams et al., 2003), pulvinar (Wilke et al., 2009), FEF (Libedinsky and Livingstone, 2011), and LPFC (Panagiotaropoulos et al., 2012). Abbreviations: BR, Binocular Rivalry; BRFS, Binocular Rivalry Flash Suppression; GFS, Generalized Flash Suppression; SfM, Structure-from-Motion; AM, Apparent Motion Quartet.

In parallel work involving human subjects, a particularly fruitful approach has employed “multistable visual stimuli,” where the same physical stimulus can give rise to multiple conscious interpretations (Leopold and Logothetis, 1999). Familiar examples of methodologies employing such stimuli include the Necker-Cube, Binocular Rivalry or Flash Suppression paradigms (Blake and Logothetis, 2002; Wilke et al., 2003; Kim and Blake, 2005; Tsuchiya and Koch, 2005) (Figure 5). Electrophysiological studies in monkeys employing such paradigms have revealed that neuronal spiking in a wide range of cortical areas is correlated with subjective perception, including retinotopically organized visual areas (Logothetis and Schall, 1989; Leopold and Logothetis, 1996; Bradley et al., 1998; Wilke et al., 2006), inferotemporal (IT) cortex (Sheinberg and Logothetis, 1997; Kreiman et al., 2002), medial temporal lobe (Kreiman et al., 2002) and fronto-parietal areas (Williams et al., 2003; Panagiotaropoulos et al., 2012). From a multitude of studies, a view has emerged that the proportion of neurons that reflect conscious perception gradually increases from early stages of visual processing (i.e. V1/V2) toward “higher-order” association areas (i.e., V4/IT) (Figure 6). Correspondingly, the few studies that have investigated spiking activity in thalamic visual nuclei have reported perceptual modulation in “second-order” (i.e., pulvinar), but not in first-order, thalamic nuclei [e.g., lateral geniculate nucleus (LGN)] (Lehky and Maunsell, 1996; Wilke et al., 2009). Interestingly, a recent study found that pulvinar neurons signaled “confidence” during motion perception judgments in a random-dot-motion paradigm, suggesting a further connection with subjective responses (Komura et al., 2013).

Animal studies have been especially informative in showing that putative NCCs of conscious content depend on the type of neural signals recorded and the type of the stimuli used (Maier et al., 2007, 2008). The most striking discrepancy has emerged in respect to early stages of visual processing, notably LGN and area V1, where neuronal firing rates did not reliably correlate with subjective perception, but where the power and oscillations in lower frequencies of local field potentials (LFP) and BOLD signals measured with fMRI apparently does reflect the perceptual state quite reliably (Lehky and Maunsell, 1996; Tong and Engel, 2001; Gail et al., 2004; Wunderlich et al., 2005; Tong et al., 2006; Wilke et al., 2006, 2009; Maier et al., 2008; Figure 6). Although we are still far from understanding the exact role of the different signal types in conscious states, there is now an appreciation of the complexity of neural signals that correlate with consciousness, other than simply firing rates of individual neurons. Thus, investigations of the coupling between electrophysiological parameters that give rise to LFPs and BOLD signals will likely deepen our understanding of the neural mechanisms underpinning consciousness (Logothetis, 2008; Sirotin and Das, 2009; Singh, 2012).

In sum, although neural correlates of conscious perception have been identified in different signal types and areas of the brain, we currently lack a comprehensive model that suggests how and when distributed network components contribute to visual consciousness. Moreover, in our opinion, we are still far from a comprehensive understanding of which components are prerequisites, substrates, or merely consequences of conscious perception, and especially, how these components work together to generate conscious percept (Aru et al., 2012; de Graaf et al., 2012). Future work in this line should therefore take advantage of network connectivity analyses (Friston et al., 2013) and perturbation methods (Massimini et al., 2009) as far as possible. Some promising work using pharmacological interventions is described below.

#### Pharmacological intervention combined with neuroimaging with non-human primate models

A promising path in monkey research is offered by the combination of pharmacological techniques with electrophysiological and functional imaging methods. For example, by reversibly perturbing neuronal activity by the local injection of various pharmacological agents while monitoring neuronal activity, it has become possible to evaluate the flow of information between contributing brain areas and to elucidate the causal contribution of a given area to conscious perception.

One productive application of this method has been in the study of spatial neglect in monkeys (Wardak et al., 2002b; Hwang et al., 2012; Wilke et al., 2012). Spatial neglect is one of the most common disorders of visual awareness (or attention) in humans, and it is characterized by impaired or lost awareness and exploration of the visual space contralateral to the lesion (Kerkhoff, 2001).

Although the advent of structural and functional MRI has identified parietal cortex, superior temporal sulcus, and posterior thalamus as critical network components in neglect patients (Karnath et al., 2001, 2002; Mort et al., 2003), even the most recent neglect models describe the networks at very gross functional levels, representing patterns of activation in whole sulci and gyri as single components of the model (Corbetta and Shulman, 2011). This lack of anatomical and functional detail is mainly due to the fact that a systematic dissection of implicated network components often proves difficult in human patient populations due to great variations in size and location of the lesions and the effects of reorganizational processes that often occur soon after lesion onset. Therefore, reversible lesions using pharmacological methods in animal models are indispensable for testing specific hypotheses. For example, the intraparietal sulcus (IPS) serves a central role in most current neglect models (Corbetta et al., 2005; Vossel et al., 2010). However, we know from monkey electrophysiological (Andersen and Gnadt, 1989; Cui and Andersen, 2007) and human fMRI studies (Grefkes and Fink, 2005) that the IPS contains a number of areas that are specialized for different modalities and effectors such as eye and hand. From recent reversible inactivation studies in monkeys we have now learned that lesions in different parts of the parietal cortex indeed evoke effector-specific spatial neglect-like symptoms (Wardak et al., 2002b; Hwang et al., 2012; Wilke et al., 2012). However, more research in animal models is required in order to develop a strictly neurobiological neglect model based on the properties of neuronal populations, rather than those of whole brain areas (Corbetta and Shulman, 2011). In addition, future research must necessarily focus on discriminating between lesion-induced changes in conscious access and changes in attention to move. This will require the development of more elaborate behavioral testing procedures, such as in (Komura et al., 2013).

#### Phylogenetic origins of consciousness

Recent years have witnessed increasing interest in the phylogenetic origins of consciousness (Edelman and Seth, 2009; Feinberg and Mallatt, 2013). Accumulating evidence indicates the presence of complex cognitive processes in animals, such as birds and certain invertebrates that are phylogenetically distant from humans. Examples include the presence of working memory, social learning, planning, and problem solving in birds (Pepperberg, 2006; Salwiczek et al., 2010), as well as the presence of rich behaviors, sophisticated learning and memory faculties in cephalopod mollusks such as the octopus (Edelman and Seth, 2009). Complex cognition may also correlates with the level of consciousness that can be achieved by an organism (e.g., Butler, 2012). Note that this is not contradictory with our previous statement that conscious perception may not be necessary for the operation of various complex cognitive processes. Even if complex cognitive processes and consciousness can be dissociated, the presence of a more complex brain, with stronger hierarchical connectivity, likely correlates with both the presence of more complex cognitive processes, and with potentially higher levels of consciousness.

While animal studies in lower vertebrates may facilitate our understanding of the phylogenetic origins of consciousness, studies of consciousness in such distant non-mammalian phyla raise the issue of distinguishing the study of primary consciousness or conscious access (in the absence of report) from that of higher order consciousness (Edelman et al., 2005). Nevertheless, in order to fruitfully pursue investigations of possible conscious states in non-human animals, it is useful to take data from humans as a benchmark and reference, comparing neuroanatomical, neurophysiological, and behavioral properties observed in non-human animals to the human case (Seth et al., 2005).

We return to future directions in this area in the section entitled, “Beyond humans and non-human primates: General issues in animal studies.”

### Theories

The increasing flow of experimental results concerning neural correlates of conscious level and conscious content in various conditions underscores the need for deep theoretical accounts to confront and hopefully integrate such a “data deluge.” Integrating theories with experiments is crucial for identifying the mechanisms that are consistently observed in association with the presence or absence of conscious contents under various levels of consciousness. Only a combination of theory and experiment can accommodate and unify the large number of results accumulated in the field within a common explanatory framework. We will now review some recent influential neuroscientific theoretical developments that attempt to link neuronal activity with conscious perception.

Block (2009) has suggested dividing current theoretical frameworks of consciousness into three categories: *biological theories, higher-order thought* theories, and *global workspace/information integration* theories. Biological theories posit that consciousness corresponds to particular biological states or processes of the brain. Candidates for such states include gamma frequency activity (Llinas et al., 1998), recurrent, or “reentrant,” processing (Edelman, 1989; Lamme, 2006), and long-distance synchrony (Melloni et al., 2007). However, to date, no consensus has been reached concerning which brain states or processes should be considered as reliable signatures of conscious processing.

According to the higher-order thought (HOT) theory (Rosenthal, 2000, 2005), a conscious experience of “red” consists of a first-order neural representation (for example, in the visual system) accompanied by a thought or higher-order cognitive state (possibly originating in frontal cortical areas) directed at that representation of “red,” with the content being the subject's experience. According to the HOT theory, consciousness implies *access* and rules out non-reportable conscious contents by theoretical fiat.

Global workspace theory (GWT) was introduced by Baars (1988) and can be viewed as a “theatre” or “arena” metaphor for mental functioning (Baars and Laureys, 2005). According to Baars et al. (2003), once conscious sensory content is established, it is distributed (broadcasted) widely to a decentralized “audience” of expert networks, including contextual and executive interpreters in parietal and prefrontal cortical areas [also see a recent thoroughly updated account in (Baars et al., 2012)]. In terms of empirical data relevant to conscious content, GWT predicts that conscious perception should mobilize widespread brain resources, while unconscious sensory processing should be much more localized (Baars, 2005). Other proposed properties of conscious perception are; (1) informativeness—that is, widespread adaptation to the novelty of the reportable signal; (2) internal consistency of conscious contents—because mutually exclusive global broadcasts tend to degrade each other (e.g., binocular rivalry); (3) interaction with an implicit self system (Baars and Laureys, 2005); and (4) limited capacity, and an operating cycle of 100–200 ms [see updated framework in Baars et al. (2012)]. Two research programs have pursued GWT in detail. A French group led by Dehaene and Changeux has focused on formal models of specific experimental paradigms that allow close comparisons between conscious and unconscious conditions. These paradigms have pinpointed the importance of widespread fronto-parietal areas activation for conscious perception, in line with the GWT notion of broadcasting (Dehaene and Naccache, 2001; Dehaene and Changeux, 2004, 2005; Dehaene et al., 2006; see also Shanahan, 2008). Franklin and his colleagues have pursued GWT in the context of a large-scale computer model of cognitive architecture and its links with working memory and attention (Baars and Franklin, 2003, 2007; Franklin et al., 2012). A recent study also showed evidence for the appearance of broadcasting with brain maturation (and presumably higher level of consciousness) in young infants (Kouider et al., 2013). However, precise empirical and mechanistic accounts of the concept of “broadcast” in the generation of conscious experience—a crucial component of GWT—are still lacking.

The information integration theory of consciousness (IITC; Tononi, 2008) has the global aim of explaining links between neuronal activity and the emergence of any conscious content. This theory emphasizes the *dynamical complexity* of conscious scenes, namely the view that conscious scenes are simultaneously integrated (i.e., united in a single percept) and differentiated (i.e., each experience rules out a vast repertoire of alternative possibilities). It predicts that a given level of consciousness will be correlated with coexisting states of differentiation and integration (Massimini et al., 2009). Recent TMS-EEG studies (Massimini et al., 2005; Ferrarelli et al., 2010; Rosanova et al., 2012; Casali et al., 2013) have indeed suggested that consciousness should necessarily require both, neuronal activity integration and differentiation. A distinguishing feature of the IITC is its attempt to explicitly link basic properties of phenomenology to neural dynamics. A challenge for the theory is precise predictions concerning which spatio-temporal scale and particular brain structures that support conscious experience have not yet been offered (Boly et al., 2009). However, several approximations of measures of information integration, accounting for these properties, are now available (Barrett and Seth, 2011; Chang et al., 2012). Related measures such as “causal density” capture a similar balance of integration and differentiation, but under a different set of mathematical assumptions, in this case emphasizing causal interactions among system elements (Seth et al., 2011a).

In the last few years, the concept of predictive coding has gained accelerating influence in the neuroscience community (Friston, 2010; Bastos et al., 2012). According to this framework, perception involves the formation of a “generative model” of sensory causes via hierarchical Bayesian inference, implying that many observable brain responses should be proportional to the mismatch between actual and predicted signals at every level of processing, i.e., to prediction errors. To date, a number of studies seem to support this hypothesis (for a review see Clark, 2013). Nevertheless, forging theoretical links between predictive coding and consciousness remains very much an open issue (Hohwy, 2012). Indeed, predictive processing could characterize both conscious and unconscious processes. For example, subjects seem to respond in a Bayesian optimal manner, even in the case of inferences concerning unconsciously processed stimuli (de Lange et al., 2011). Furthermore, predictive coding has been suggested to take place even at the level of motor reflexes in the spinal cord through the fulfillment of proprioceptive predictions (Friston, 2012). On the other hand, predictive coding has also been invoked to account for the subjective feelings of agency (Friston, 2012) and, in a separate line, for both subjective feeling states (emotions) (Seth and Critchley, 2013), and the sense of subjective reality of the world and the self (i.e., “presence”) (Seth et al., 2011b; Critchley and Seth, 2012). Future conceptual and experimental work must be directed toward understanding the links between Bayesian brain inference and conscious perception.

## Future directions

### Experimental approaches

Despite the substantial advances summarized above, no consensus has yet been reached on the nature or location(s) of the NCC or NCCs, for either conscious content or conscious level. As mentioned previously, not only is this is a necessary step in realizing the basic science of consciousness, but such a consensus carries important clinical implications for the design of consciousness markers applicable to conditions like the vegetative state or general anesthesia.

#### Human consciousness

Research on altered states of consciousness would greatly benefit from the acquisition of data using highly conserved paradigms and neuroimaging modalities in different states such as coma, general anesthesia, sleep, seizures, and somnambulism (sleepwalking, or NREM sleep parasomnia). A deeper understanding of the neural correlates of the LOC during sleep or general anesthesia is also urgently required, as well as relations between neural processes and altered conscious states evoked by pharmacological manipulations [e.g., psylocibin; (Carhart-Harris et al., 2012)].

Most studies investigating neural correlates in these states merely compare brain functions under general anesthesia, sleep, or vegetative state with those apparent in normal wakefulness. However, these apparently straightforward comparisons may be misleading. For example, it is now known that subjects awakening from general anesthesia report a significant incidence of dreaming [26%, (Mashour et al., 2011)]. Results from studies using the isolated forearm technique (performing sedation without paralyzing the forearm, allowing for a probing of response to command) also report a median incidence of responsiveness of about 37% during general anesthesia under perioperative conditions (Sanders et al., 2012). To better understand these observations, studies comparing brain activity under general anesthesia with or without dreaming, as well as with or without response to commands, are crucial (Sanders et al., 2012). A better understanding of differences in brain activity during sleep in the presence or absence of dream report after awakening is also needed. In vegetative state patient populations, comparing brain activity in subjects with or without a response in active volitional paradigms is critical (Owen, 2013). Finally, a more refined distinction between neural correlates of unconsciousness (the absence of any conscious contents) vs. neural correlates of disconnectedness (the absence of perception of the environment) during both sleep and general anesthesia is essential (Sanders et al., 2012).

Current efforts also aim at validating clinical “consciousness-meters” that would enable us to detect language-independent (Majerus et al., 2005; Scott et al., 2011; Casali et al., 2013) minimal signs of awareness (of any content) in non-communicative patients. If signs of consciousness are detected, then improvements in brain-computer interface technology will permit some of these “functionally locked-in” patients to express their thoughts and wishes (for a review, see Chatelle et al., 2012). Advances in hardware, software, and statistical signal analysis and modeling of brain recordings from portable EEG or functional near-infrared spectroscopy devices will be needed to achieve an acceptable accuracy in these technologies at the single subject level. Avoiding both false negative (i.e., failing to detect signs of consciousness or communication in aware subjects) and false positive results (i.e., detecting consciousness or communication when there is none) is especially challenging in the absence of a gold standard for conscious awareness (Laureys and Boly, 2008). In addition, new prognostic tests continue to improve our capacity to predict the probability of recovery of consciousness (Jox et al., 2012). Again, these tools will need to show their clinical value at the single patient level.

Scientifically, the cellular mechanisms which underlie the functional recovery of awareness after severe traumatic or anoxic brain injury remain obscure; axonal sprouting, neurite outgrowth, neurogenesis (known to occur predominantly in associative cortical areas in non-human primates), or even apoptosis are being tested as candidate processes (Laureys et al., 2006). The challenge still remains to identify the conditions in which, and the mechanisms by which, some patients with disorders of consciousness may recover.

Regarding consciousness research in healthy awake humans, further research must be undertaken to uncover the precise dissociations observed between consciousness and cognitive functions such as attention, cognitive control, and volition, either taken alone or in association with one another (Boly and Seth, 2012). These functions need to be carefully and independently manipulated from conscious contents to isolate the neuronal correlates of consciousness without contamination of dissociable cognitive functions (Koch and Tsuchiya, 2007; van Boxtel et al., 2010; Watanabe et al., 2011; Bor and Seth, 2012).

The functional advantages of conscious over non-conscious perception remain to be elucidated. In this regard, paradigms dissociating the effects of conscious perception from those of better behavioral performance are critical (Lau and Passingham, 2006; Persaud et al., 2011). The question of conscious and unconscious is not limited in perception; it remains unclear to what extent our actions are triggered by conscious freewill and preceded by unconscious processes (Soon et al., 2008).

#### Issues in non-human primate studies

What have we learned from non-human primate studies about the neural mechanisms of visual conscious perception within the past half-century and what remains to be investigated? Amazingly enough, the fundamental question of whether activity in the early visual cortex is a part of the neural substrate of a conscious percept, or merely an enabling factor, has not yet been answered (Crick and Koch, 1995). Are loops between visual and fronto-parietal areas necessary to enable a conscious percept, and do second-order thalamic nuclei such as the pulvinar contribute to consciousness by facilitating communication in those cortical networks (Sherman and Guillery, 2002; Saalmann and Kastner, 2011)? Several laboratories have now begun to investigate these questions by combining brain inactivation/stimulation with electrophysiological and functional imaging methods.

Another vexing issue of equal importance is the nature of the neural computations that underlie visual awareness. Simply stated, does consciousness rely only on neuronal firing rates, or on the precise timing of population activity (Fries et al., 1997; Tallon-Baudry, 2003; Gail et al., 2004; Wilke et al., 2006; Uhlhaas et al., 2011)? To tackle this issue experimentally, one would wish for methods that allow the systematic manipulation of neuronal timing while keeping firing rates constant (Stopfer et al., 1997).

Another important question is whether specific cell types (e.g., deep vs. superficial pyramidal cells, Von Economo neurons, etc.) are more important for consciousness than others? Do consciousness-related neurons have specific morphologies and/or molecular properties, or is their connectivity the critical factor (but see Maier et al., 2007)? These questions can, in theory, be tackled by means of newly emergent technologies, such as optogenetics, that through genetic modification of ion channels allow the manipulation of the electrical activity of specific neuronal phenotypes by activating them with light (Deisseroth, 2011; Yizhar et al., 2011). The targeted expression of genetically encoded proteins, together with an appropriate readout, has enormous potential to unravel the microcircuitry (at micro-/millimetric scales) underlying conscious perception. However, time will tell whether the light-mediated, optogenetic control of neural activity—with its limited spatial coverage—represents the best means of manipulating consciousness-related activity in large primate brains (Diester et al., 2011; Gerits et al., 2012; Nielsen et al., 2012).

Finally, in order to improve the credibility of non-human primate models of human consciousness, we need to deepen our understanding of similarities and differences in the underlying neural networks. This can be achieved by conducting more comparative studies that employ the same behavioral tasks and measurement methods (e.g., fMRI) in both human and non-human species (Kagan et al., 2010).

#### Beyond humans and non-human primates: general issues in animal studies

The investigation of possible conscious states in animals presents a number of daunting challenges, as well as some important opportunities. Most significant among the challenges are: (1) the assessment of consciousness in the absence of verbal report; (2) the characterization of brain structures and neurophysiological signatures—perhaps homologous to those already identified in mammals and birds—that might support conscious states in lower vertebrates (Karten, 2013); (3) the identification of neuroanatomical and neurophysiological properties that might suggest at least the possibility of conscious states in invertebrates with relatively large, differentiated, and centralized nervous systems (i.e., cephalopod mollusks, arachnids); and finally (4) the development and application of more elaborate neuroimaging and electrophysiological techniques applicable to a wide variety of small non-primate (and even aquatic and/or invertebrate) species.

The absence of verbal report for the assessment of consciousness in the majority of non-human phyla is not an insurmountable issue. As has been shown in a number of studies of non-human primate models of blindsight and spatial neglect, training regimes can be employed that require animals to give a behavioral report of their putative experiences (Stoerig and Cowey, 1995; Wilke et al., 2012). Such “metacognitive commentaries” provides a robust complement to neuroanatomical, electrophysiological, and psychophysical techniques in the study of NCC in non-human primates. In addition to subjective reports, there are also statistical and objective criteria that are now frequently employed to obtain behavioral correlates of consciousness in animal studies (Leopold et al., 2003).

Similar neuronal mechanisms might support a level of consciousness across mammalian species in general, given brain structures and circuitry that are closely homologous to the human case, conserved neurophysiological signatures during sleep states and general anesthesia, and comparable behavior (Edelman et al., 2005; Edelman and Seth, 2009). Beyond mammals, however, this argument is more difficult to make, primarily owing to an incomplete understanding of the relevant neuroanatomy and neurophysiology. Nevertheless, with technical advances in microscopic reconstruction and the use of neurogenetic markers, it is increasingly clear that many elements of brain structures considered critical for supporting conscious states in mammals (e.g., the six-layered neocortex) are conserved more deeply in the vertebrate lineage, most notably among birds (Reiner et al., 2005; Karten, 2013). As well, avian electrophysiological signatures have been identified that are reminiscent of mammalian sleep (Rattenborg et al., 2009). These advances, along with accumulating evidence for sophisticated learning and memory faculties (most notably among the corvids; see Emery and Clayton, 2004; Bugnyar and Heinrich, 2006), suggest that the rigorous study of avian consciousness represents a plausible near-future scenario. The same applies as well to studies of lower vertebrates such as reptiles, amphibians, and even fish, given rapidly advancing neurophysiology and neuroanatomy in these species.

Investigating consciousness in invertebrate phyla is more challenging. We lack detailed connectivity maps in all but a few invertebrates. Nevertheless, there are invertebrates—most notably the cephalopod mollusks (e.g., octopus, cuttlefish, and squid) and arachnids (e.g., jumping spiders)—that show rich behavioral repertoires suggestive of high-order neural function [e.g., learning, memory, integration, and monitoring; see (Fiorito and Scotto, 1992; Biederman and Davey, 1993; Edelman et al., 2005)]. Whether this level of neural function includes conscious processes remains very much an open question (but see Mather, 2008). In any case, such a question remains important in potentially shedding substantial light on possible universal properties of consciousness, as well as its evolutionary trajectory on Earth.

### Theories of consciousness

In order to consolidate the results of the many relevant experiments that have been conducted within a single conceptual framework, theories of consciousness must become more precise and generate experimentally testable predictions. Accomplishing this requires both additional conceptual work from theorists and greater knowledge of brain architecture and neural computations relevant to consciousness, in order to guide and constrain theory development. In particular, it is unclear how differential computations performed by different cortical layers, cell types (e.g., inhibitory vs. excitatory), various neuromodulators, and connections (e.g., feedforward vs. feedback, driving vs. modulatory) contribute to consciousness. The causal roles of cortex, thalamus, and brainstem remain poorly understood in this context. These and similar considerations underline the need to reconcile existing theories (especially those based on integrated information and complexity) with specific and well grounded anatomical and neurodynamical observations.

More global challenges for theoretical perspectives involve elaborating the links between predictive processing and conscious perception (Hohwy, 2012), and unifying these accounts with those based on integrated information and/or dynamical complexity. Theoretical developments are also needed to characterize the relations between phenomenal consciousness and access conscious (to the extent that there is a difference), and, in a related direction, to better our understanding how metacognition and first-order conscious contents are associated. Here, a promising direction may involve the elaboration of signal-detection theory frameworks to include top-down signal processing (Barrett et al., 2013). As this research develops it will integrate with other areas not discussed in this paper, notably the neural bases of intention, agency, and conscious selfhood. Overall, theoretical developments will help move from simple correlation between neural events and conscious level and content, toward causal and explanatory accounts that show how specific neural mechanisms give rise to specific aspects or dimensions of conscious phenomenology (Seth, 2010). In doing so, theoretical developments must also engage with the importance of distinguishing NCCs *per se* from their prerequisites and consequences (Aru et al., 2012; de Graaf et al., 2012).

### Methodological advances

For the sake of empirical rigor, new theories must be accompanied by simultaneous methodological advances. Recent biotechnological developments offer great promise in furnishing the appropriate data. New technologies, such as large-scale electron microscopy and brain slices histology (Chung et al., 2012, 2013) and 2-photon microscopy of the cortex in behaving animals (Prakash et al., 2012), combined with optogenetics (Deisseroth, 2011; Yizhar et al., 2011), light-sheet microscopy (Ahrens et al., 2013), cortical micro-electrode recordings (Du et al., 2011), and optical imaging studies (Harvey et al., 2012) will soon enable us to better establish how the brain's micro-scale anatomy is coupled to functional properties and information processing *in vivo*. Large-scale projects such as the one initiated at the Allen Institute for Brain Research are likely to be decisive developments in this realm (Callaway, 2011). The combination of micro-scale and macro-scale brain anatomy with functional data using large-scale computational models could shed important light on brain dynamics and neural computation present in various brain states, such as those during sleep, general anesthesia, or various cognitive tasks.

Furthermore, the appearance of new techniques such as laminar and columnar fMRI [enabled by recent progress in high field scanner development and fast sampling pulse sequences, (Feinberg et al., 2010; Zimmermann et al., 2011; Van Essen et al., 2012; Chen et al., 2013)], coupled to high density intracranial electrophysiology recordings (Viventi et al., 2011) should also yield a considerable amount of information about brain processes linked to conscious perception in animals and humans.

Significant advances have also been made in the genetic manipulation of non-human species (Venken et al., 2011). In the realm of primate research, great interest has recently been generated by the use of a primate subspecies, the marmoset, as a “biomedical supermodel” for genetic manipulation (Sasaki et al., 2009). Marmosets have a very high rate of reproduction and a short maturation time, biological attributes that lend themselves nicely to paradigms combining genetics and neurophysiology. These new world monkeys are capable of performing various complex cognitive tasks that could conceivably be tested in various paradigms analogous to those employed in human experiments (Remington et al., 2012). As well, they present a relatively flat and smooth cortex that is extremely amenable to electrophysiological investigations.

Returning finally to the human case, the first complete genetic mapping of a human brain has recently been made available to the research community, carrying enormous promise for a better understanding of genotypic vs. phenotypic interactions in the brain (Hawrylycz et al., 2012). As burgeoning new techniques and experimental opportunities continue to generate an ever-increasing flow of valuable data, the development of robust theoretical and mechanistic accounts will be even more crucial in crafting a coherent and plausible account of the links among consciousness, genotype, phenotype, and brain function.

### Conflict of interest statement

The authors declare that the research was conducted in the absence of any commercial or financial relationships that could be construed as a potential conflict of interest.
